# The French CONSTANCES population-based cohort: design, inclusion and follow-up

**DOI:** 10.1007/s10654-015-0096-4

**Published:** 2015-10-31

**Authors:** Marie Zins, Marcel Goldberg, CONSTANCES team

**Affiliations:** Population-based Epidemiological Cohorts Unit, Inserm UMS 011, 16 Avenue Paul Vaillant Couturier, 94807 Villejuif, France; Versailles-Saint Quentin University, Versailles, France

**Keywords:** Population-based cohort, Chronic diseases, Aging, Socioeconomic factors

## Abstract

The CONSTANCES general-purpose cohort is intended to serve as an epidemiological research infrastructure accessible to the epidemiologic research community with a focus on occupational and social factors, and on chronic diseases and aging. CONSTANCES will also provide useful public health information to the public health authorities since it was designed as a large representative sample of the general French adult population. CONSTANCES is designed as a randomly selected representative sample of French adults aged 18–69 years at inception; 200,000 subjects will be included over a five-year period. At inclusion, the selected subjects are invited to complete questionnaires and to attend a Health Screening Center (HSC) for a comprehensive health examination. A biobank will be set up. The follow-up includes a yearly self-administered questionnaire, and a periodic visit to an HSC. Social and health data are collected from the French national databases. Data collected for participants include social and demographic characteristics, socioeconomic status, life events, behaviors, and occupational factors. The health data cover a wide spectrum: self-reported health scales, reported prevalent and incident diseases, long-term chronic diseases and hospitalizations, sick-leaves, handicaps, limitations, disabilities and injuries, healthcare utilization and services provided, and causes of death. To take into account non-participation at inclusion and attrition throughout the longitudinal follow-up, a cohort of non-participants was set up and will be followed through the same national databases as participants. Inclusion begun at the end of 2012 and more than 82,000 were already included by September 2015. A public call for nested research projects was launched.

## Background

Research on the causes of diseases in the field of environmental, occupational, social, genetic or pharmacoepidemiology often reveals small relative risks for individual risk factors. Very large-scale cohorts, providing high quality phenotyping and long-term follow-up, are required to ensure sufficient statistical power to better understand the role of various personal and environmental factors and their interaction with complex genetic traits. For instance, known associations between genetic variants and chronic diseases show typical allelic odds ratios in the range 1.1–1.4 [1]. The reliable identification of such effects demands vast data sets [2]. Case–control studies show that thousands of cases are required even when interest focuses on the simplest situations, and when the research question focuses on the study of gene-environment and gene–gene interactions and the comprehensive exploration of causal pathways, tens of thousands of cases will often be required. Tens of thousands of subjects may also be required to study a quantitative phenotype (e.g., measured blood pressure), because allelic effect sizes may be as small as one-tenth of a standard deviation, or even less [3]. Beginning with the Framingham Study, which follows-up from 1948 on a few thousand volunteers [4], much larger prospective cohorts including hundreds of thousands of subjects were launched in different countries, such as the Nurses’ Health Study [5], the One Million Women Study [6], the UK Biobank [7], the Kadoorie Study of Chronic Disease in China [8], the Norwegian CONOR Consortium [9], the EPIC European Prospective Investigation into Cancer and Nutrition [10] or LifeLines in the Netherlands [11]. Other very large population-based cohorts with hundreds of thousands of participants are currently being implemented in different countries, such as the German National Cohort [12], LifeGene in Sweden [13], or the Cartagene Cohort in Québec, Canada [14].

## Main objectives

The overarching objective of the CONSTANCES project is to establish a large population-based cohort to contribute to the development of epidemiologic research. It was designed as a general-purpose prospective cohort intended to serve as an open epidemiological research infrastructure accessible to the scientific community for conducting ancillary projects on a variety of research questions. It will serve as an important scientific instrument, in a similar manner to a telescope or a particle accelerator, for example, built not to answer a specific question but rather to help analyze a wide range of scientific problems. In this regard, the design of CONSTANCES relied on the experience of the GAZEL Cohort Study, an open general-purpose prospective cohort established in 1989 by our research group which is currently supporting more than 80 different nested research projects on very diverse scientific topics [15–17]. Although designed as a general-purpose cohort intended to host nested projects with a very broad scope, we have focused on specific areas. We are especially interested in occupational and social factors, on chronic diseases and aging. Regarding occupational factors, CONSTANCES should contribute to the study of occupational exposure in the etiology of cancer and in exploring the genetic polymorphisms that make individuals susceptible to these factors [18]. Musculoskeletal disorders in relation to working conditions and biomechanical and psychosocial factors at work are also a key topic of interest, focused on the short and long-term medical, social and professional major consequences of musculoskeletal disorders [19]. The effects of exposure to occupational chemicals on chronic respiratory diseases [20] and on neurodegenerative diseases [21] and cognitive functioning is an important concern. Psychosocial factors at work contribute to coronary heart disease [22], depression and mental health [23] and other outcomes. Due to the economic context in industrial countries, there is also a major interest in workability and other determinants of early exit from the labor force, as well as on determinants and consequences of extending working life [24]. Social determinants of health inequalities are another major area of interest for CONSTANCES. This covers social inequalities in the occurrence, treatment and socioeconomic consequences of common conditions such as diabetes, cancer, depression and other psychiatric problems or cardiovascular diseases [25]. Aging is a major challenge in all industrialized countries, but studies are essentially limited to the age groups above 65 years and provide little information about earlier life periods [26], even though factors that lead to impairments, disabilities, and chronic diseases at advanced ages often begin early in life, and they continue to accumulate throughout life. CONSTANCES should contribute to the study of many research questions about aging, such as the study of the occupational, personal and genetic determinants of cognitive decline, the effects of retirement on cognition, or factors that may lead to inactivity and isolation, factors and mechanisms that contribute to successful aging, and conversely those that contribute to disabilities and/or frailty [27]. Efforts are also to be made to understand the causes of individual and social heterogeneity in aging by investigating the nature of the association between risk factors and cognitive aging in terms of cumulative risk, risk trajectories or critical period models [28]. Research on consequences of aging is focused on the impact of poor functional status on survival and functioning and the potential causes of its variation by socioeconomic position [29].

The second main objective of the CONSTANCES cohort is to provide useful public health information to the public health authorities and health care regulatory bodies in order to contribute to a better knowledge of the health and health care resource utilization of the French population. For this purpose CONSTANCES was designed as a large representative sample of the general French adult population, characterized by a broad coverage of health problems and health determinants.

## Design

### Cohort composition

Considering our objectives, CONSTANCES was designed as a sample representative for age, gender and socioeconomic status (SES) of the French adult population aged 18–69 at inception. However, due to our partnership with the National Health Insurance Fund administered by the “Caisse Nationale d’Assurance Maladie des travailleurs salaries” (CNAMTS), we had to restrict the source population of CONSTANCES to salaried workers, professionally active or retired and their family (more than 85 % of the French population, i.e. approximately 50 million people), thus excluding agricultural and self-employed workers which are affiliated to other health insurance funds.

We plan to include 200,000 participants over a 5-year period. As CONSTANCES is a general-purpose cohort, we assessed the potential of CONSTANCES to conduct epidemiologic studies likely to have good statistical power. We estimated the number of some major health outcomes expected in the CONSTANCES cohort over a moderately long term in a cohort with an age and sex structure identical to that of the French general population aged 18–69 years at the last available national census. Table 1 presents the number of expected events at the end of 5, 10, and 15 years for events for which we have reliable national reference data [30–32]: deaths and incidence of cancer, ischemic heart disease, and Alzheimer disease. For these major outcomes, the number of these serious events is high and will make possible numerous studies with satisfactory power.

**Table 1:** Expected number of incident major health outcomes during the follow-up of the CONSTANCES cohort

	5-year follow-up			10-year follow-up			15-year follow-up		
	Men	Women	Total	Men	Women	Total	Men	Women	Total
Death, all causes	4131	2133	6264	9727	5502	15,229	16,983	10,736	27,719
Incident cases of ischemic heart disease (35–64 years)	681	138	819	1418	290	1708	2178	452	2630
Incident cases of Alzheimer disease	265	240	505	793	1007	1800	1548	2469	4018
Incident cancers	3162	2220	5381	7036	4855	11,892	11,444	7823	19,267
Lip, oral cavity, pharynx	306	47	353	644	103	747	1005	165	1170
Colon-Rectum	357	251	608	817	590	1407	1360	1011	2370
Liver	106	16	121	232	36	268	373	60	433
Pancreas	54	34	88	121	78	199	197	133	330
Larynx	89	7	96	189	16	205	298	26	324
Lung	502	91	593	1093	199	1292	1743	321	2064
Melanoma	66	86	152	139	180	319	216	278	494
Breast	0	900	900	0	1922	1922	0	3031	3031
Uterus	0	100	100	0	220	220	0	357	357
Ovary	0	91	91	0	196	196	0	312	312
Prostate	689	0	689	1627	0	1627	2755	0	2755
Bladder	166	22	188	380	55	435	633	98	730
Kidney	113	55	168	245	123	367	390	198	589
Thyroid	19	66	85	39	134	173	58	201	259

French national reference data come from [30–32]

### Procedures for inclusion

**Fig. 1:**
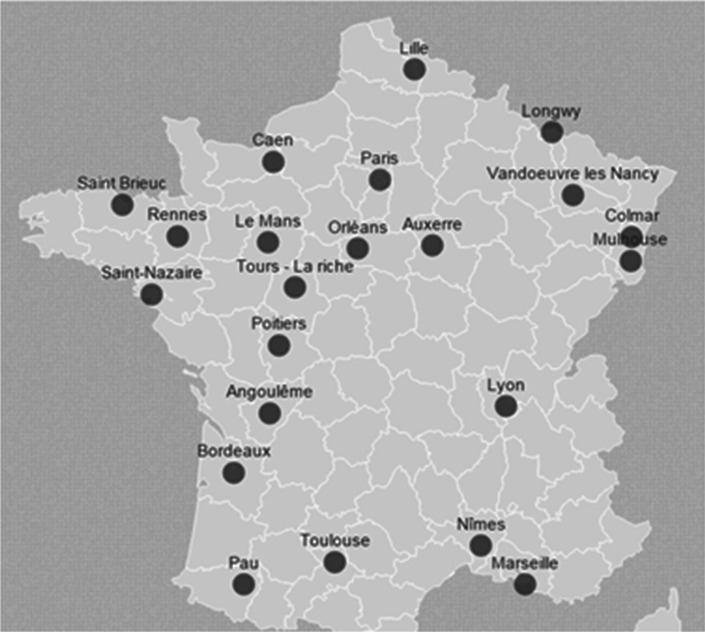
Geographical location of CONSTANCES recruitment centers in France

In France, everyone with health insurance from CNAMTS, as well as their dependents, is entitled to receive free health examinations that include extensive work-ups conducted in Health Screening Centers (HSCs). Overall the 110 HSCs located in all France conduct approximately 500,000 health examinations annually. Thanks to our partnership with CNAMTS, we are including the cohort participants in 22 selected HSCs located in 19 “départements” in different regions of France (Fig. 1).

Randomly selected eligible persons (see below) receive at home an invitation to come to their HSC. We selected HSCs that have experience with the recruitment of large numbers of people and with participating in epidemiological studies. All are large, have a staff motivated to work in epidemiology, and use advanced medical equipment; their geographic distribution represents the principal regions of France. We are proceeding gradually to the establishment of the entire cohort which will last over a 5-year period. Inception started at the end of 2012, and the final cohort will be constituted by the end of 2017.

Those who volunteer receive questionnaires to complete at home before attending their HSC where they sign an informed consent and benefit for a health examination.

### Procedures for longitudinal follow-up

Participants are followed-up through “active” procedures (implying them directly). An annual self-administered questionnaire is completed by the subjects at home, using either a paper questionnaire or internet. They will also be invited every 5 years for a new health examination in a HSC. Maximizing their personal participation rate is essential. Accordingly, regular contact with participants includes a CONSTANCES Cohort Journal, which will present results, nested projects, etc., and is sent regularly to participants. A website was also created (www.constances.fr). The subjects included in CONSTANCES are also followed up “passively” (so-called because this follow-up does not require the subjects’ participation) by annual linkage with three national social and health data databases.

The National Retirement Insurance Fund administered by CNAV ensures the retirement pension for every individual in France who had health insurance from CNAMTS at least once during his or her life. CNAV has therefore set up a system that allows it to collect social and occupational data from different organisms and schemes that manage various forms of insurance and other social protection. The CNAV regularly receives for its databases employers’ annual reports (occupation, salary), and information about periods of employment and unemployment from social welfare organizations (e.g., sick leave, maternity leave, unemployment, and diverse social benefits) [33].

The National Health Insurance Fund administered by CNAMTS manages the SNIIRAM database which covers the entire French population [34]. The SNIIRAM contains exhaustive individual medical detailed data from different sources: reimbursement data (doctors and other health professionals visits, prescribed drugs, medical devices); so-called “long-term diseases” (serious chronic diseases exempt from co-payments and user fees); hospital discharge records, including for each hospitalization principal and associated diagnoses, medical and technical procedures. Table 2 shows the main data extracted from the SNIIRAM database.

**Table 2:** Main data extracted from the SNIIRAM national database

Recipient
Gender
Date of birth
Area of residence
Disability pension
Occupational injury
Benefits
Nature of the benefit (drugs, health professional visits, vaccination…)
Drug and medical device codes
Period
Date of treatment start and end
Hospitalization start date
Accident date
Prescription date
Recipient medical information
Disease codes
Presumed pregnancy start
Tooth
Performing and prescribing healthcare professional
ID, age, category, medical specialty
Activity type for non-physicians
Type of practice
Performing and prescribing professional establishment
Establishment number and category
Hospital medical data
Date and mode of entry and of exit
Total duration of stay
Weight at birth
Primary diagnosis
Secondary diagnoses
Severity indicator
Medical procedures
Cause of death/transfer code

Finally, vital status and causes of death are obtained from the National Death Registry-CepiDc [30].

### Principal data collected from different sources

Here we summarize the main data to be collected from different sources (questionnaires, medical examination, national health and social databases), at each stage of the study; the detailed English version of the inclusion and follow-up data catalog can be downloaded from CONSTANCES’ website [35, 36].

#### Social and demographic characteristics

Social position, educational and income level, employment and marital status, household composition, socioeconomic status of parents and spouse, material living conditions (type of housing, household income, etc.), including geocoding of the residency address.

#### Health

Personal and family disease history; self-reported health scales (perceived health, quality of life, mental health, and specific scales for cardiovascular, musculoskeletal, and respiratory diseases); incident and prevalent diseases (from self-reports, social security long-term diseases and hospital discharge); sick leaves, handicaps, limitations, disabilities and injuries and healthcare utilization and management; and date and cause of death. In the HSC examination weight, height, waist-hip ratio, blood pressure, electrocardiogram, vision, hearing, and lung function, laboratory tests (blood sugar level, lipid work-up, liver function tests, blood creatinine levels, complete blood cell counts, urine tests) are measured.

#### Behaviors

Smoking and alcohol consumption, dietary habits and physical activity, cannabis use, sexual orientation.

#### Occupational factors

From questionnaires: lifelong and current occupational exposure to chemical, physical, and biological agents; postural, mechanical and organizational constraints; stress at work. Full job histories are coded for linkage with available job-exposure matrices developed by the Occupational Health Department of the National Institute for Health Surveillance [37].

#### Specific health problems of the elderly (45 years and older)

CONSTANCES collects detailed data on cognitive and physical performance from the age of 45, which is earlier in life than most of the available cohorts [38]. Neuropsychologists proceed to an evaluation of functional capacities: Instrumental Activities of Daily Living (IADL) scale [39]; cognitive functions are assessed through the MMSE [40], trail making A–B [41, 42], Wechsler’s coding subtest [43]; digital finger tapping test [44], word fluency, formal lexical and semantic evocation [45, 46], RL/RI-16 memory test [47, 48]; physical functioning assessment includes walking speed [49], balance [50] and hand grip tests [51].

#### Biobank

Due to budget restrictions we plan to collect biological samples (blood and urine) from only half of the cohort (n = 100,000) starting in 2016 during inclusion visits to the HSC of the new participants. For blood, we will store, total blood, plasma aliquots on EDTA plasma separator tube (PST), and plasma aliquots on Hep Li PST, serum aliquots (dry serum separator tube). For urine, we will keep aliquots. Standardized procedures for biological samples collection will be used, including standardized blood sampling (pre-treatment of the samples in each recruitment center within 30 mn after the collection), transport from each site to the central laboratory within the night (<24 h) at 4–8 °C, robotized aliquoting in cryotubes (2D barcodes) in the central biorepository, and storage in vapor phase nitrogen; sample retrieval will be automated. In addition to this basic biobanking program, CONSTANCES will offer optional programs for specific research projects on subsets of participants, in which additional samples such as washed erythrocytes, RNA, proteins, mononuclear cells, saliva, or hair and nails may be collected.

### Quality control and validation of health events

The self-administered questionnaires undergo the standard verifications: percentages of non-response, missing data, delay in return, etc.

For standardizing data collected in the study centers, we developed a quality program, including quality assurance and quality control procedures in order to obtain high quality medical examination data. We first organized working groups composed of personal from participating sites (MD and nurses), epidemiologists and quality assurance specialists supervised by experts of each domain concerned by the data measured or collected, in order to develop standard operating procedures (SOP), which detail the measurement method for each type of data. The SOPs also describe the materiel admissible for the study, the required annual certification, the periodic verifications or maintenance (all SOPs can be downloaded from the CONSTANCES website: http://www.constances.fr/espace-scientifique/pos.php). For each measurement, all steps of the realization were detailed in order to minimize the inter-operator variation. Prior inclusion, we performed a physical inspection of each site and each site’s employee involved in the study has been trained prior participation. The two first days of inclusions, a monitor was present on site to support the study staff. Any study site representative involved in the study has to be trained by a monitor. After training the practice of the trainees are regularly followed by a monitor in order to minimize drifts over time. Practices are monitored on site on a monthly basis. Practice of each study site member is followed at minimum once per year.

The quality control process includes a validation plan, tracking the missing data, the out of ranges or any warning waiting for predefined consistency check. Each month the data exported from the site and imported in the CONSTANCES database are sorted out from the database to perform quality controls. When discrepant data are encountered, the monitor identifies the origin of the discrepancy and the concerned data have to be corrected and reintegrated into the CONSTANCES database. For each category of data, the monitor identifies the source document (where the data has been recorded for the first time) and verifies the consistency between the site data and the CONSTANCES database extraction. Finally, we perform a permanent statistical monitoring of the inter-operator and inter-site variability.

As medical data extracted from the national databases are not always accurate, particular attention is paid to validation of the diagnoses extracted from the health-related administrative databases, which are routinely verified using a specific procedure. Potential cases are first identified from the available sources: self-reported diseases in the annual follow-up questionnaire, diagnoses extracted from the SNIIRAM database (“long-term diseases”, hospital diagnoses). Participants having given a specific consent for being contacted or for contacting their doctor or hospital (97.4 % of the participants gave such a consent) are then contacted by telephone. A short questionnaire allows for confirming that the person really reported a disease and for collecting additional data (date of the occurrence of the disease for instance); the participant is asked to send medical documents allowing for collecting specific data and for diagnosis validation (pathology report, electrocardiogram…). When the subject cannot be reached or when he/she cannot provide the documents, the hospital or the general practitioner is contacted. Finally, the cases are adjudicated by specialized expert committees. Initially, we are particularly focusing on some major outcomes: cardiovascular events, cancers, and neurodegenerative diseases.

### Data management

CONSTANCES’ data are centralized in a unique database stored in a highly secured environment. We describe here the main features of the data management process. The first step is sampling of the eligible people within the CNAV database (see below); after sampling, identification data (names, postal addresses, telephone numbers) are encrypted and kept by a thrusted third party, an independent organization in charge of sending the invitations to participate to CONSTANCES; thus only a study number is stored in the central CONSTANCES database. After the inclusion of the participants in the study medical centers, several data streams (where the participants are identified by their study number) coming from multiple sources feed regularly the database using different media: paper questionnaires are computerized; data from the study centers are sent to the CONSTANCES database through internet or by postal mail depending on their nature; data extracted from the administrative databases are encrypted and sent to CONSTANCES by internet by CNAMTS and CNAV; inclusion questionnaires are completed on paper, while for follow-up questionnaires participants can chose between paper or internet using a specific internet platform. Consent forms are stored by the thrusted third party. For the validation of the diagnoses, as it implies a direct contact with the participants and/or their doctor or hospital, the thrusted third party send the contact data (names, telephone, consent forms) to an external telephone platform.

The CONSTANCES database itself is divided into two parts. Raw data from the different sources are stored in a working database where different controls and data cleaning are performed and the raw data are archived. Cleaned data are then transferred into the study database, which is used for research. Safety is ensured by various provisions (encryption of data, passwords, regular back-ups…). Encrypted data are transferred to external researchers for their studies by internet; data potentially allowing identification of the participants are impoverished (zip code instead of address, age group instead of date of birth, etc.).

The data management system of CONSTANCES was approved by CNIL, the national data protection authority.

### Periodicity of follow-up

The periodicity of follow-up varies according to the sources. A self-administered mail questionnaire is sent annually, thus allowing close follow-up, by collecting numerous data without asking subjects for too much work each year. At the same time, it will facilitate rapid response for setting up new studies and establish a sense of loyalty in the participants; too long a delay between two questionnaires is a factor that promotes dropping out [52]. Some data will be collected annually (health status and reported morbidity, life events and characteristics of place of residence, smoking, alcohol, etc.), while others will be collected at longer intervals, according to a planned calendar (health scales and questionnaires for a specific health area or specific risk factors). Because the national databases essentially record events continuously, the follow-up of the data they provide will be permanent. Finally, participants will also be asked to come to the HSC every 5 years for medical and laboratory examinations.

### Control of selection effects

Selection effects are one of the major sources of bias in epidemiologic surveys. They can bias estimates of disease prevalence or incidence (or of prevalence of exposure to a risk factor) and of associations between exposures and diseases of interest. In longitudinal cohorts, selection effects may occur at inclusion and throughout follow-up because of cohort attrition.

The problem of biases linked to selection effects is very different depending on whether the objectives are analytic or descriptive [53]. In a cohort whose inclusion procedures are the same for all subjects (the case of CONSTANCES), in principle the exposure-disease relation does not differ between subjects who are included and those who are not [54–56]. Therefore, the selection procedures at inception for CONSTANCES participants should generate minimal bias in analytic studies, although the observed exposure effect relationships may be affected if the highest exposures are underrepresented, which often happens for factors like alcohol or tobacco consumption. On the other hand, the problem of attrition during follow-up may cause substantial bias if the probability of continued follow-up is different in exposed and unexposed subjects or in those who do or do not become ill, as it is often the case [57].

For descriptive studies of the frequency of health problems and exposures, the parameters of interest must be estimated in a representative sample of the target population. In this regard, the potential concerns for CONSTANCES are mainly incomplete geographical coverage of the recruitment centers and factors associated with voluntary participation. We have verified that the structure of the population of the “départements” where the CONSTANCES HSCs are located is essentially identical to that for France as a whole for the principal demographic, social, and occupational characteristics; we should thus be able to generalize the CONSTANCES results to the French population (data not shown).

Using volunteer subjects inevitably produces selection effects, even in studies that use random drawing from an appropriate sampling base, as it is the case of CONSTANCES, as eligible individuals may refuse participation (become non-participants), a potential source of bias. To compensate, researchers usually attempt to collect a minimum data set for the non-participants (mainly age, sex, and social category), to facilitate subsequent adjustments for estimating the relevant parameters. This approach nonetheless has some limitations. First, it is not always possible to collect the adjustment data for non-participating subjects. Nor is it always clear whether these data are sufficient to control for potential bias, because we know, for example, that within the same socioeconomic category there are many important differences in terms of health, behavior, lifestyles, social networks, etc. [58, 59]. Finally, it is rarely possible to control completely for potential selection bias because it is rare to have the relevant data collected simultaneously for the participants and the non-participants.

To obtain a representative sample of the target population and to minimize the biases associated with selection effects at inclusion and during follow-up in CONSTANCES, we took the following steps. The sampling base at inclusion is composed of all persons aged 18–69 years and covered by CNAMTS in the catchment areas of the CONSTANCES HSCs. Sampling is done within the CNAV database which includes exhaustively all the persons in France affiliated to the CNAMTS. The random drawing is stratified according to unequal inclusion probabilities, based on data from participation in previous surveys involving invitations to HSCs [60]. We also drawn a “parallel cohort” from a random sample of 400,000 non-participants for whom we prospectively collect data from the same national databases than for the participants: social and occupational characteristics (sex, age, work status, occupation, social category), through the CNAV database, health and health-care utilization from the SNIIRAM and the National Death Registry. Auxiliary data extracted from CNAV and SNIIRAM cover three years before inception for both the participants and the sample of non-participants. We are thus able to estimate the probabilities of participation in CONSTANCES associated with sociodemographic and health variables using logistic regression models, to compute weights for correcting unit nonresponse and to estimate adjusted prevalence of questionnaire variables. As in epidemiological surveys auxiliary health and social data are usually not available for non-respondents, this approach has rarely been used to correct the prevalence estimates for nonresponse bias, with few exceptions which proved to effectively correct for nonresponse [61].

A major concern of long-term prospective cohorts is attrition, potentially inducing biases and affecting the power of the study [52]. We can assume that almost none of the people included in CONSTANCES will be permanently lost to follow-up, since the participants will be followed passively through the SNIIRAM, CNAV and National Death Registry files. There will nonetheless be attrition due to the failure to return the annual questionnaire. Coefficients of adjustment for attrition are calculated by a method similar to the one used to calculate the coefficient of adjustment for initial non-participation based on the data collected at inclusion for participants as well as the SNIIRAM and CNAV data.

## Advancement

After a field pilot during a four to five-month period in seven centers, including about 3500 subjects [62], the recruitment started in late 2012. Currently (September 2015), more than 82,000 participants are included in the cohort. The participation rate to the annual follow-up questionnaire of subjects who were included in 2012, 2013 and 2014 was higher than 80 % each year.

The preliminary analysis of the available data showed that this sample is close to the general population of adults in France regarding the main socioeconomic variables (Table 3).

**Table 3:** CONSTANCES cohort: main sociodemographic characteristics of the sample

	%
Age	
18–29	11.3
30–39	17.0
40–49	22.1
50–59	23.7
60+	25.8
Gender	
Men	46.1
Women	53.9
Education	
No diploma or lower than high school	27.4
High school	16.6
College	23.5
University	30.5
Missing	2.0
Marital status	
Single	23.7
Married, civil partnerships	60.1
Divorced, separated	10.9
Widower	2.4

Data available in July 2015 (n = 57,922)

There was quite a diverse distribution of occupations and working conditions, lifestyle factors, and the prevalence rates of various diseases and symptoms were close to those from other available French surveys (data not shown).

We also verified that the use of auxiliary data from national databases on both respondents and non-respondents for correcting unit nonresponse and to estimate adjusted prevalence of questionnaire variables was efficient. As shown in Table 4, reweighting techniques used in a previous work [61] proved to meaningfully improve the estimates of prevalence in the population of different conditions related to health and behaviors. When adjusting only for stratification variables (age, sex and socioeconomic status), changes were observed in the expected direction reflecting the classical underrepresentation of people with a poor health status (low self-rated health, alcohol abstinent which are often persons having a severe disease) or overweighed, and the over participation of ex-smokers. We further adjusted on health data extracted from the SNIIRAM database for both participants and non-participants which were associated with the probability of participation in logistic regression models (disability, chronic diseases and hospital discharge diagnosis, number of visits to a doctor, expenses for ambulatory care). The changes observed with the first adjustments were markedly amplified, yielding more accurate estimates of the prevalences, showing that adjusting only on “classical” parameters (age, sex and socioeconomic status) as it is sometime done in health surveys, is not sufficient if not completely misleading, as it is the case for persons having a fasting plasma glucose >7 mmol: adjustment on the stratification variables resulted in a decrease of prevalence, reflecting the high participation of aged subjects, while adjustment on diagnosed diabetes led on the contrary to a strong increase of prevalence reflecting the low participation of persons suffering from this condition.

**Table 4:** Crude and adjusted prevalence of selected reported conditions (percentage, 95 confidence intervals and percentage of change after adjustment)

	Crude prevalence	Adjusted on stratification variables (1)	Further adjustment on health variables (2)
Low self-rated health	20.1 (18.0–22.2)	21.8 (19.3–24.2) (+8.5 %)	23.6 (20.6–26.6) (+17 %)
Ex-smoker	28.6 (26.2–30.1)	26.0 (23.5–28.4) (−10 %)	25.2 (22.4–28.0) (−12 %)
Alcohol abstinent	18.3 (16.3–20.3)	20.5 (18.0–22.9) (+4 %)	21.3 (18.3–24.2) (+16 %)
Obesity	10.1 (8.5–11.6)	10.8 (9.0–12.7) (+7 %)	12.3 (9.8–14.7) (+21 %)
Fasting plasma glucose >7 mmol	2.2 (1.5–3.0)	2.1 (1.3–2.6) (−5 %)	3.5 (2.0–5.0) (+59 %)

1—Age, sex and socioeconomic status

2—Disability, chronic diseases and hospital discharge diagnosis, number of visits to a doctor, expenses for ambulatory care

## Research in CONSTANCES

Every group in France or in other countries, public or private, is entitled to apply to develop a nested project within CONSTANCES and to access to its database. Projects are evaluated by the CONSTANCES Scientific Committee on feasibility and scientific quality criteria. A Charter describes the rules that have been established for using the CONSTANCES infrastructure, regarding legal aspects, data confidentiality and security, ethics, access to the database in the case where only available data are required or when the collection of supplementary data directly from the cohort participants is needed, as well as sharing of these supplementary data, access to the biological and genetic material, responsibilities of the CONSTANCES infrastructure and of external groups, dissemination of data and results, publications and authorship, acknowledgments, follow-up of the project and funding. The material needed for applying can be downloaded from the CONSTANCES website (http://www.constances.fr/index_EN.php#propose).

A first call for proposals was launched in 2014 among a restricted set of French investigators who collaborated in the preparation of the protocol of CONSTANCES. More than 40 projects covering a wide range of topics were proposed and approved by the Scientific Committee; the list of accepted projects is available at: http://www.constances.fr/espace-scientifique/projets-valides.php. In May 2015, the public call for ancillary projects proposals was launched; it is planned that the Scientific Committee will examine the applications twice a year.

## Discussion

Considering its large size, the extensive coverage of the French adult population, the wealth of data collected from different sources, and its openness to the scientific community, the CONSTANCES cohort should constitute a powerful tool for public health information and epidemiologic research in many different fields.

CONSTANCES has several strengths. It was designed both to help answer research questions in diverse areas and to provide public health information needed by the health authorities. To facilitate the latter aim, we devised a specific sampling scheme including a non-participants cohort and developed complex statistical procedures in order to take into account selection effects at inception as well as during the follow-up of the cohort. Once completed CONSTANCES will be a large cohort, including persons living and working in diverse settings, from large cities to small villages in different regions of France, with a broad range of socioeconomic status and trades. Numerous data are collected at inception, including an extensive medical, physiological and biological examination, and a large biobank will be set up. The follow-up is extensive, relying both on active participation of the volunteers through annual questionnaires and regular visits to the HSCs, and on passive methods through the regular linkage to health and socioeconomic national exhaustive databases. Of particular importance is the high frequency of measurements from many different sources, allowing for analyses of life course trajectories of health in relation to personal, social, occupational factors and major life events. Specific efforts were put into the quality of data collection and the validation of main outcomes in order to provide a highly phenotyped cohort. A unique feature of CONSTANCES is also to include a comprehensive set of cognitive and physical tests starting as young as 45 years, which is earlier in the life course than most available studies on ageing.

The CONSTANCES has also some limitations. Due to the voluntary participation of cohort members, there will probably be an underrepresentation of hard-to-reach subjects, such as heavy drinkers or socially excluded persons. Comparisons between participants and non-participants at inclusion and during the follow-up through the “non-participants cohort” allow assessment of potential biases due to selection effects, but lack of sufficient numbers in some categories might be a problem. Even more importantly, despite its large size CONSTANCES will not offer sufficient power to study rare outcomes or exposures. Simulations under several hypotheses regarding the prevalence of exposure and expected relative risk and duration of follow-up since inception, showed that in most of the situations where the relative risk is below 2, especially when interactions have to be taken into account, power will be satisfactory after at least 5 years of follow-up for situations where the annual incidence of the outcome is over 10/100,000 and the prevalence of exposure over 10 % (data not shown). This limit is common to all longitudinal cohorts, which is why CONSTANCES participates in the Biobanking and Biomolecular Resources Research Infrastructure-Large Prospective Cohorts (BBMRI-LPC) consortium for networking of prospective studies in Europe [63].
